# Biomarker Discovery of Acute Coronary Syndrome Using Proteomic Approach

**DOI:** 10.3390/molecules26041136

**Published:** 2021-02-20

**Authors:** Miji Shin, Sang Hyun Park, Sora Mun, Jiyeong Lee, Hee-Gyoo Kang

**Affiliations:** 1 Department of Senior Healthcare, Graduate School, Eulji University, Seongnam 13135, Korea; miji_shin@naver.com (M.S.); sora6456@naver.com (S.M.); 2 Department of Internal Medicine, School of Medicine, Eulji University, Daejeon 34824, Korea; psh@eulji.ac.kr; 3 Department of Biomedical Laboratory Science, School of Medicine, Eulji University, Uijeongbu 11759, Korea; 4 Department of Biomedical Laboratory Science, College of Health Sciences, Eulji University, Seongnam 13135, Korea

**Keywords:** acute coronary syndrome, biomarker, diagnosis, proteomics

## Abstract

Acute coronary syndrome (ACS) is a condition in which the coronary artery supplying blood to the heart is infarcted via formation of a plaque and thrombus, resulting in abnormal blood supply and high mortality and morbidity. Therefore, the prompt and efficient diagnosis of ACS and the need for new ACS diagnostic biomarkers are important. In this study, we aimed to identify new ACS diagnostic biomarkers with high sensitivity and specificity using a proteomic approach. A discovery set with samples from 20 patients with ACS and 20 healthy controls was analyzed using mass spectrometry. Among the proteins identified, those showing a significant difference between each group were selected. Functional analysis of these proteins was conducted to confirm their association with functions in the diseased state. To determine ACS diagnostic biomarkers, standard peptides of the selected protein candidates from the discovery set were quantified, and these protein candidates were validated in a validation set consisting of the sera of 50 patients with ACS and 50 healthy controls. We showed that hemopexin, leucine-rich α-2-glycoprotein, and vitronectin levels were upregulated, whereas fibronectin level was downregulated, in patients with ACS. Thus, the use of these biomarkers may increase the accuracy of ACS diagnosis.

## 1. Introduction

Acute coronary syndrome (ACS) is used to describe the clinical symptoms or signs induced via acute myocardial ischemia, including unstable angina (UA), non-ST segment elevation myocardial infarction (NSTEMI), and ST segment elevation myocardial infarction (STEMI) [1]. Atherosclerotic plaque formation in the coronary artery, due to the accumulation of low-density lipoprotein (LDL), increases the number of macrophages and T-lymphocytes, thereby increasing stress of the fibrous cap and matrix degradation, reducing the matrix synthesis, and inducing a plaque rupture [2]. Exposure of the ruptured plaque to blood causes the formation of a thrombus that accumulates in the coronary artery, and this leads to a blockage of the blood supply, and ultimately induces the development of ACS [3]. Additionally, ACS has a poor prognosis since a thrombus can induce an infarction, partially or completely blocking the blood supply and potentially leading to the occurrence of myocardial necrosis [3,4]; hence, ACS is a disease with extremely high morbidity and mortality worldwide [5].

Accurate ACS diagnosis is essential for effective treatment and for improving the survival rate [6]. Current diagnostic methods focus on confirming specific changes, such as an increase or decrease of the ST segment using an electrocardiogram (ECG), or evaluation of biomarkers, such as creatinine kinase-myocardial band (CK-MB), troponin I, and troponin T [7,8], and are thus limited. Although ECG examination is typically used to diagnose the existence of heart disease, its sensitivity is only 50% [9], while biomarkers, such as troponin T, do not reach appropriate levels to enable an accurate diagnosis following symptom onset [9,10]. Therefore, due to the diagnostic limitations of the biomarkers currently available, guidelines now recommend immediate perfusion therapy for patients exhibiting increasing ST segment in ECG examination and showing hemodynamic instability or persistent ischemic chest pain regardless of changes in biomarkers [11]. Furthermore, since chest pain, a typical symptom of ACS, is a common symptom in other diseases, it is difficult to diagnose ACS in patients who report the development of chest pain [10]. Finally, UA cannot be diagnosed using existing biomarkers, which are typically used to diagnose the existence of myocardial necrosis [12]. Therefore, the identification of novel ACS diagnostic biomarkers with high specificity and sensitivity is warranted.

To identify new biomarkers, previous studies have targeted proteins since proteins, unlike genes, include information regarding modifications, such as alternative splicing and posttranslational modifications (PTMs), directly involving proteins in the functions of an organism [13,14,15]. Therefore, proteins influence disease onset and progression. Proteomics, the study of the identification, structure, and in vivo function of the proteome [15], can help to identify biomarkers associated with the diagnosis, stage, prognosis, and treatment of diseases [16,17,18]. To this end, mass spectrometry has been primarily used to analyze complex in vivo proteomes owing to its high sensitivity in analysis [19,20]. Additionally, this technique has been widely used, not only for ACS, but also for various other diseases, such as cancer [21,22,23,24,25].

**Figure 1:**
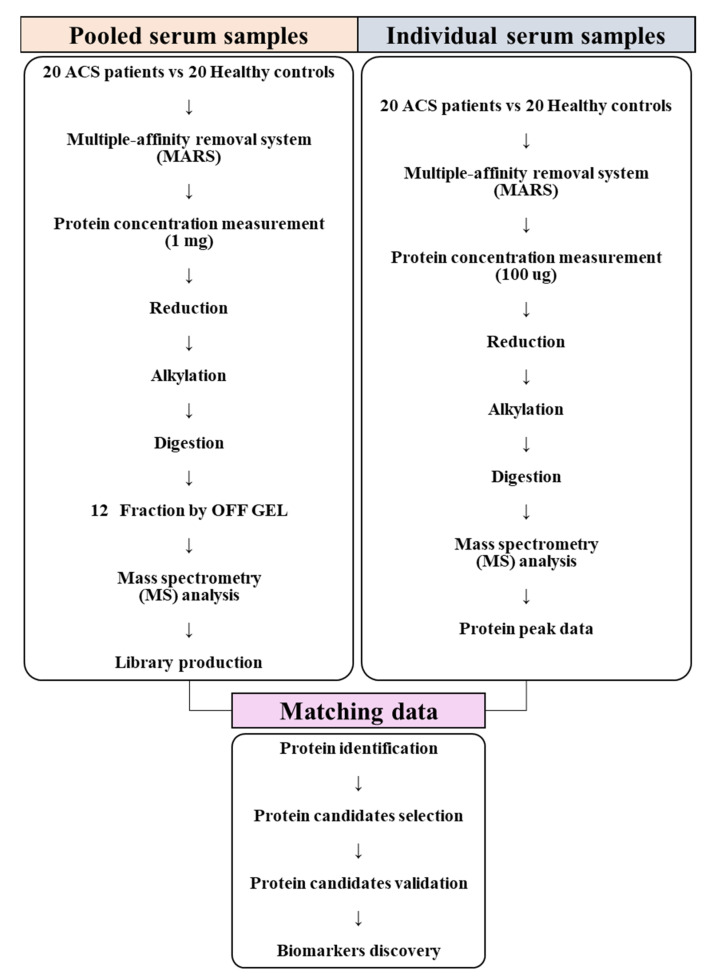
Flowchart of the experimental setup. Serum samples, consisting of individual and pooled serum samples, were collected from 20 patients with acute coronary syndrome (ACS) and 20 healthy controls and prepared for conducting mass spectrometry analysis. Pooled serum samples were analyzed using the information-dependent acquisition (IDA) mode to generate a library. Individual serum samples were analyzed using the sequential window acquisition of all theoretical fragment-ion spectra (SWATH) mode. Data produced using the SWATH mode were matched with the library for protein identification. After identifying the statistically significant proteins among all identified proteins, functional analysis was conducted to confirm the processes and functions associated with these proteins in ACS. Diagnostic biomarkers for ACS were selected through validation of the selected protein candidates.

To identify novel ACS diagnostic biomarkers, this study was performed to analyze the serum samples of patients with ACS and healthy controls using mass spectrometry, to identify proteins that significantly differed between the two groups, and to proceed with functional analysis to confirm the association between proteins and the occurrence of ACS. Then, the selected protein candidates were analyzed via quantification and validation of their levels in the serum samples using the multiple reaction monitoring (MRM) acquisition mode (Figure 1).

## 2. Results

### 2.1. Identification and Confirmation of Differentially Expressed Proteins between Patients with ACS and Healthy Controls

**Figure 2:**
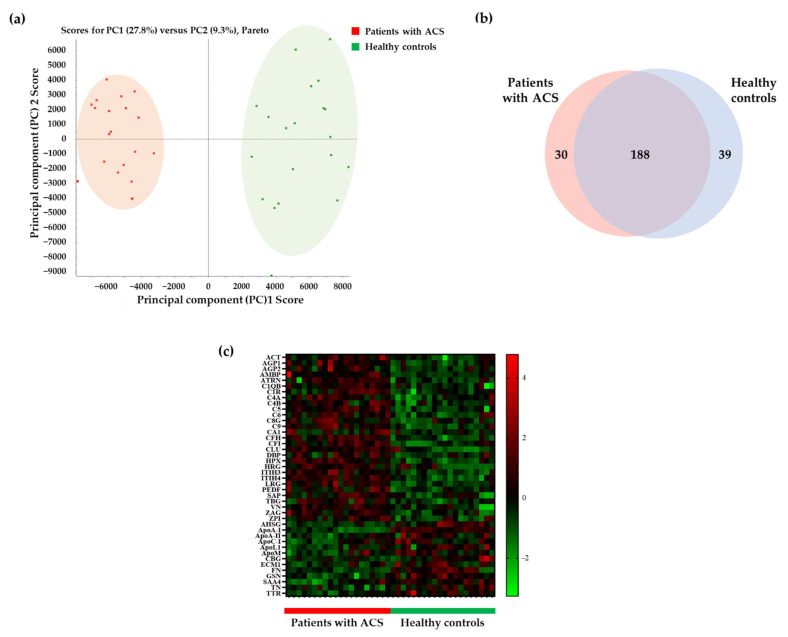
Selection of differentially expressed proteins between patients with ACS and healthy controls. (**a**) The principal component analysis (PCA) score plot shows the separation of samples of patients with ACS and healthy controls. (**b**) The Venn diagram shows confirmed commonly expressed proteins between patients with ACS and healthy controls. The number of proteins identified in patients with ACS and healthy controls were 218 and 227, respectively, with the number of proteins identified in both groups being 188. (**c**) Of the 188 proteins, proteins with *p*-value < 0.05 and a fold change of 1.2 or more or 1/1.2 or less were selected using the *t*-test. We selected 42 proteins exhibiting significant intensity of peak and false discovery rate (FDR) in the heat map. ACT, α-1-antichymotrypsin; AGP1, α-1-acid glycoprotein 1; AGP2, α-1-acid glycoprotein 2; AMBP, protein AMBP; ATRN, attractin; C1QB, complement C1q subcomponent subunit B; C1R, complement C1r subcomponent; C4A, complement C4-A; C4B, complement C4-B; C5, complement C5; C6, complement component C6; C8G, complement component C8 γ chain; C9, complement component C9; CA1, carbonic anhydrase 1; CFH, complement factor H; CFI, complement factor I; CLU, clusterin; DBP, vitamin D-binding protein; HPX, hemopexin; HRG, histidine-rich glycoprotein; ITIH3, inter-α-trypsin inhibitor heavy chain H3; ITIH4, inter-α-trypsin inhibitor heavy chain H4; LRG, leucine-rich α-2-glycoprotein; PEDF, pigment epithelium-derived factor; SAP, serum amyloid P-component; TBG, thyroxine-binding globulin; VN, vitronectin; ZA2G, zinc-α-2-glycoprotein; ZPI, protein Z-dependent protease inhibitor; AHSG, α-2-HS-glycoprotein; ApoA-I, apolipoprotein A-I; ApoA-II, apolipoprotein A-II; ApoC-I, apolipoprotein C-I; ApoL1, apolipoprotein L1; ApoM, apolipoprotein M; CBG, corticosteroid-binding globulin; ECM1, extracellular matrix protein 1; FN, fibronectin; GSN, gelsolin; SAA4, serum amyloid A-4 protein; TN, tetranectin; TTR, transthyretin.

We obtained data after the analysis of pooled and individual serum samples using the information-dependent acquisition (IDA) and the sequential window acquisition of all theoretical fragment-ion spectra (SWATH) modes, respectively. We matched these data, for protein identification, and identified 257 matched proteins. Additionally, we confirmed that each group was clustered in the principal component analysis (PCA) score plot (Figure 2a). We found that 188 proteins were commonly expressed between 218 proteins identified in patients with ACS and 227 proteins identified in healthy controls (Figure 2b). To confirm differentially expressed and statistically significant proteins, we conducted the *t*-test for all 188 proteins and selected 99 proteins (*p*-value <0.05 and fold change (patients with ACS/healthy controls) ≥1.2 or ≤1/1.2). Moreover, among these 99 proteins, we selected 42 proteins with peptides exhibiting over 1000 peak intensity and less than 1% false discovery rate (FDR) in PeakView. The lower the FDR value, the more reliable the process of matching the data of the samples and the library for protein identification. These 42 proteins are illustrated in the heatmap (Figure 2c) and include 29 upregulated and 13 downregulated proteins in patients with ACS (Table S1 in Supplementary Materials).

### 2.2. Functional Analysis of Differentially Expressed Proteins between Patients with ACS and Healthy Controls

**Figure 3:**
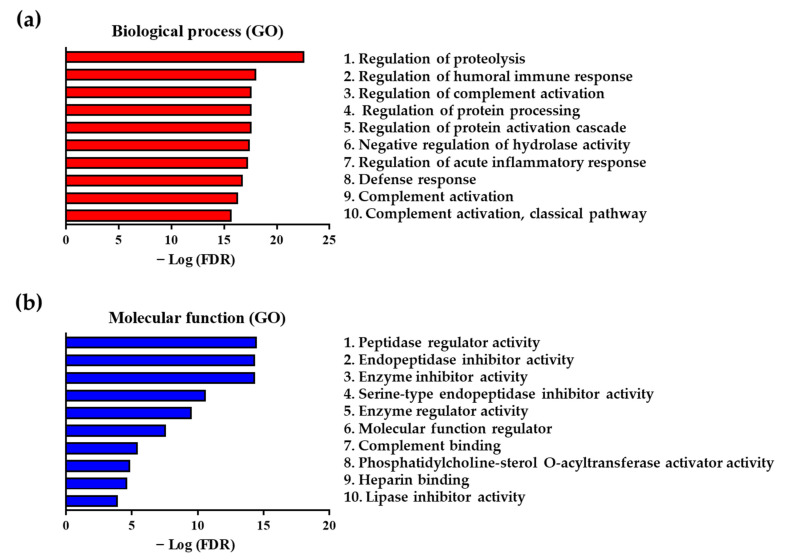
Functional analysis of 42 differentially expressed proteins between patients with ACS and healthy controls. (**a**) The top 10 biological processes (gene ontology; GO) and (**b**) molecular functions (GO) associated with differentially expressed proteins between patients with ACS and healthy controls.

We further confirmed the processes and functions related to the differentially expressed and statistically significant 42 proteins by performing biological process (gene ontology; GO) and molecular function (GO) analyses (Figure 3). We conducted the functional analysis of the 42 proteins using the STRING database. The lower the FDR, the more the proteins corresponded to those that constituted the function; data on the proteins were used as input into the STRING database for conducting functional analysis. Our analysis of biological processes (GO) showed that the regulation of proteolysis ranked first, with the lowest FDR of 2.25 × 10^−23^%, among all processes, followed by the regulation of humoral immune response (FDR = 8.57 × 10^−19^%) and regulation of complement activation (FDR = 2.37 × 10^−18^%), which were ranked second and third, respectively (Figure 3a and Table S2 in Supplementary Materials). Our results of the molecular function (GO) analysis showed that the peptidase regulator activity (FDR = 3.20 × 10^−15^%), endopeptidase inhibitor activity (FDR = 4.37 × 10^−15^%), and enzyme inhibitor activity (FDR = 4.47 × 10^−15^%) were ranked first, second, and third, respectively, showing a lower FDR compared with that of other ranks of molecular function (Figure 3b and Table S3 in Supplementary Materials).

### 2.3. Selection of Protein Candidates for Identification of ACS Diagnostic Biomarkers

We selected peptides for protein candidates by filtering data with conditions of the MRM acquisition mode. Finally, of the forty-two proteins, we selected seven candidates as potential ACS diagnostic biomarkers with good sensitivity and specificity and validated them using the MRM acquisition mode (Table 1).

### 2.4. Discovery of ACS Diagnostic Biomarkers

**Figure 4:**
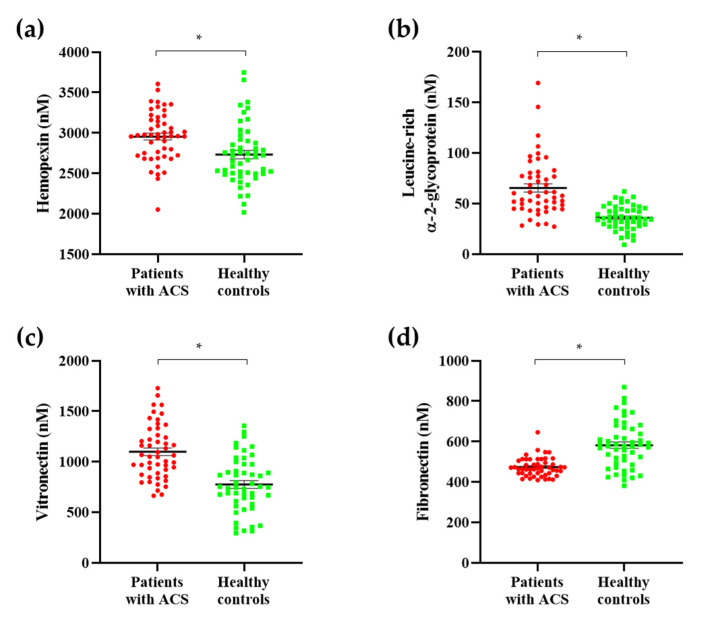
Scatter plots of ACS diagnostic biomarkers. To identify ACS diagnostic biomarkers, we developed a validation set. Among the protein candidates, ACS biomarkers were selected as proteins showing the same expression in the discovery set. Among the protein candidates, expression levels for (**a**) hemopexin, (**b**) leucine-rich α-2-glycoprotein, and (**c**) vitronectin were upregulated in patients with ACS. (**d**) Fibronectin expression was downregulated in patients with ACS. All ACS diagnostic biomarkers showed statistically significant results. * *p* < 0.001.

**Figure 5:**
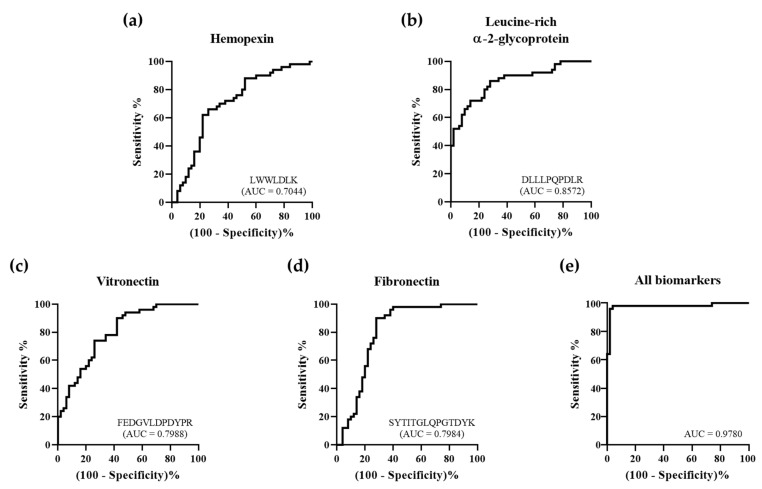
Receiver operating characteristic (ROC) curves of ACS diagnostic biomarkers. To identify ACS diagnostic biomarkers, we developed a validation set. The area under the curve (AUC) of (**a**) hemopexin was 0.7044, (**b**) leucine-rich α-2-glycoprotein was 0.8572, (**c**) vitronectin was 0.7988, and (**d**) fibronectin was 0.7984. (**e**) When using the entire set of discovered biomarkers as a panel, the AUC was 0.9780.

The seven protein candidates were validated by quantification using the MRM acquisition mode. We evaluated all parameters suitable for the quantification of each standard peptide and selected only one parameter in each standard peptide for quantification (Table 2). We obtained quantitative results for these protein candidates through the analysis of the scatter plots and receiver operating characteristic (ROC) curves. Among these proteins, expression levels for hemopexin, leucine-rich α-2-glycoprotein, and vitronectin were upregulated in patients with ACS, whereas fibronectin expression was downregulated, as illustrated by scatter plots, resulting in the same expression in the discovery set (Figure 4). Additionally, analysis of the ROC curves of these four protein candidates showed a significant area under the curve (AUC) value of 0.7 or more. Particularly, the AUC was 0.9780, which was extremely significant when using the entire set of discovered biomarkers as a panel. (Figure 5). Based on these findings, we selected these four protein candidates as potential ACS diagnostic biomarkers.

## 3. Discussion

ACS pathogenesis is induced by the accumulation of LDL (which primarily consists of cholesterol) in the intima of arteries, as well as its subsequent reaction with free radicals in the blood, leading to its oxidation, i.e., ox-LDL. Conversion of LDL to ox-LDL is known to create an inflammatory environment, since induced macrophages remove ox-LDL, ingest the cholesterol accumulated by LDL, and are then converted to foam cells, finally forming plaque [26,27]. In addition to macrophages, smooth muscle cells (SMCs) and phagocytic cells are induced into fibrous caps [26]. These cells are known to synthesize and secrete enzymes, such as matrix metallopeptidases (MMPs) and cysteine proteinases to induce the degradation of the extracellular matrix (ECM) and apoptosis of SMCs and endothelial cells. As a result, the unstable plaque is ruptured, and thrombus is formed in the arteries, which can induce necrosis of the myocardium [28], thereby placing ACS in the list of diseases with high morbidity and mortality worldwide [5]. Early and accurate diagnosis, as well as prompt treatment of ACS are important for reducing the morbidity and mortality associated with the disease. Troponin, the most widely used biomarker, has extremely high sensitivity and specificity in the diagnosis of myocardial infarction; however, it exhibits low specificity within 3 h of symptom onset [9,10]. Additionally, troponin level has been found to be elevated in not only acute myocardial infarction (AMI), but also in other diseases and complications that cause myocardial damage, such as myocarditis and stress-induced cardiomyopathy [10]. Studies have shown that troponin may also be useful in the diagnosis of AMI associated with ACS, but not UA [12]. Therefore, the need for new biomarkers for ACS remains unmet. In our study, we used mass spectrometry to identify and validate ACS diagnostic biomarkers.

Hemopexin (HPX) is a scavenger protein with high affinity for free heme present in the blood; HPX transports heme to macrophages and hepatocytes and excess heme production is suppressed via the catalytic action of heme oxygenase [29]. HPX is synthesized in the liver following an inflammatory reaction; an increase in HPX levels promotes the expression of the nuclear factor-κB (NF-κB) pathway, with HPX acting as a positive acute-phase protein having anti-inflammatory effect [29,30]. Particularly, by removing heme in atherosclerosis, HPX has been demonstrated to suppress the secretion of proinflammatory cytokines from macrophages, thereby illustrating a protective function for high-density lipoprotein (HDL) in the serum [31]. Since the level of apolipoprotein A-I (ApoA-I) that constitutes part of HDL has been found to be decreased by the action of MMP-8 (Table S1 in Supplementary Materials) [32], HDL levels may be decreased. To mitigate this effect, HPX would suppress the oxidation of HDL, thus enhancing the antiatherogenic, antioxidant, and anti-inflammatory properties of HDL [33,34,35]. The level of HPX was also reported to be increased in patients with unstable plaque; we confirmed the increased levels of HPX in patients with ACS [36].

Leucine-rich α-2-glycoprotein (LRG), a biomarker for various inflammatory diseases [37,38], is primarily synthesized and secreted from endothelial cells, neutrophils, macrophages, and hepatocytes after induction of inflammation [39,40,41]. The increased level of LRG is associated with advanced differentiation of T-lymphocytes [38]. T-lymphocytes are known to secrete granzyme for the degradation of ECM components, such as collagen and fibronectin [28]. Furthermore, T-lymphocytes secrete perforin and granzyme to induce apoptosis in target cells [42]. Therefore, LRG may induce plaque rupture through matrix degradation and apoptosis. Parallel with our results, it has been reported that the level of LRG is increased in patients with ACS [43].

Vitronectin (VN), a glycoprotein mainly synthesized in the liver, is present in the serum, ECM, and α-granules of platelets [44]. Since VN has a multiple binding domain, it has been reported to be involved in several functions, such as platelet adhesion and aggregation, coagulation, fibrinolysis, peripheral proteolysis, complement-dependent immune response, cellular attachment, and migration [45]. Moreover, it has been shown that during the plaque-induced inflammatory reaction, secreted cytokines induce the production of VN [41] that establishes interaction with coagulation and fibrinolysis-related proteins and binds to and stabilizes the plaque-induced plasminogen activator inhibitor-1 (PAI-1), a protein that suppresses the conversion of plasminogen to active plasmin [41]. The decomposition of the intra-arterial thrombus is suppressed by the action of the PAI-1/VN complex, which suggests that the decomposition of thrombus produced by the ruptured plaque would also be suppressed [46,47]. Additionally, VN can promote cell adhesion via the ανβ3 integrin in the cell membrane and ECM, which is hindered by PAI-1 to inhibit cell proliferation and induce apoptosis [46,48]. Similar to our findings, it has been reported that the level of VN is increased in patients with cardiac artery disease (CAD) [44,49], and that PAI-1 may bind to VN and function as a complex, with both of their levels being simultaneously increased in the plaque [50].

Fibronectin (FN) is a glycoprotein and a major member of ECM. Increases in the level of FN have been reported to activate endothelial cells during the production of plaque in coronary arteries, with substances in the plaque, such as ox-LDL, transmitting NF-κB signaling and inducing proinflammation, during the initial stage [51]. However, during the late stage of plaque formation, the induced inflammatory response has been shown to result in the release of proteases, such as chymase, tryptase, granzyme, and MMP-7 from inflammatory cells, resulting in ECM degradation and FN fragmentation [32,52,53]. Similar to our findings, the levels of FN have been found to decrease in patients with STEMI [54,55].

These novel biomarkers could be assessed via demonstration of their role in the pathogenesis of ACS, from the initial accumulation of cholesterol-containing LDL in the arteries, to the induction of inflammation, formation of plaque, and the release of cytokines from inflammatory cells. Both interleukin (IL) and tumor necrosis factor (TNF) produced by inflammatory cells have been shown to cause an accumulation of SMCs and lipids in plaques, thereby inducing the production of proteases, such as MMP, and conversely suppressing the expression of tissue inhibitor of metalloproteinase (TIMP), an enzyme inhibitor of MMP [56]. Furthermore, we also demonstrated an MMP-mediated degradation of the matrix components, such as ECM collagen and elastin, and FN decomposition and reduction [53]. Our functional analysis showed that pathways, such as proteolysis regulation, protein processing, protein activation cascade (Figure 3a), and peptidase regulator activity (Figure 3b), were prominently expressed in patients with ACS. Therefore, we confirmed that the significantly differentiated proteins between patients with ACS and healthy controls were associated with matrix degradation. As mentioned, LRG1 levels increased following inflammation, thereby inducing matrix degradation and apoptosis [28,41,43]. Additionally, it has been reported that MMP-8 also decomposes ApoA-I, with reduction in the activity of HDL that is primarily composed of ApoA-I. Finally, the reverse cholesterol transport by HDL was shown to be disrupted [57]. As shown in Table S1 in Supplementary Materials, the level of ApoA-I was decreased. It was also confirmed that VN, induced by the inflammatory reaction, induced apoptosis and suppression of thrombus degradation through binding to PAI-1 [50]. Since the results showed that 42 proteins subjected to functional analysis were associated with serine-type endopeptidase inhibitor activity, the proteins were therefore associated with the activity of PAI-1, a serine protease inhibitor (Figure 3b). Meanwhile, the activity of HPX, which is known to protect HDL, has been shown to increase [31].

In this study, we assessed differentially expressed proteins between patients with ACS and healthy controls to investigate and obtain new ACS diagnostic biomarkers. Then, we conducted functional analysis using statistically significant proteins and confirmed the processes and functions associated with inflammation and protein degradation, which are key steps involved in the pathogenesis of ACS. Hence, these proteins were considered to be associated with ACS. We further advanced our validation by quantifying the seven most significant protein candidates and finally selected four candidates, with an AUC of 0.7 or higher, as ACS diagnostic biomarkers. Additionally, when using these biomarkers as a panel for diagnosis, the AUC was 0.9780. ACS, including UA, which cannot be diagnosed using necrotic biomarkers, can be accurately diagnosed using these biomarkers. These novel biomarkers can help to better investigate the pathogenesis and diagnosis of ACS, and further investigations are warranted to verify and establish the application of these biomarkers in clinical practice.

## 4. Materials and Methods

### 4.1. Subjects and Collection of Serum Samples

Serum samples from patients with ACS and healthy controls were provided by Eulji University Hospital in accordance with the procedures approved by the Institutional Review Board (EMC 2016-03-019, 31 March 2016). Our discovery set cohort included 20 patients with ACS and 20 healthy controls. The number of subjects in the validation set cohort was 50 patients with ACS and 50 healthy controls (Table 3). Patients with any other disease except for ACS were excluded from the study, whereas healthy controls were selected based on the absence of CAD. Whole blood samples from patients with ACS and healthy controls were collected in vacutainers without anticoagulant. Then, serum samples were obtained by centrifugation at 4000× *g* and 4 °C for 5 min and stored at −70 °C until use.

### 4.2. Preparation of Serum Samples

Samples consisted of pooled serum samples and individual serum samples. Both pooled serum and individual serum samples were diluted in a 1:3 ratio using the multiple-affinity removal system (MARS) buffer A (1.2% sodium chloride and 0.02% sodium azide in high-performance liquid chromatography (HPLC)-grade water; Sigma-Aldrich, St. Louis, MO, USA) and centrifuged at 15,000× *g* for 1 min using a membrane filter (Spin-X Centrifuge Tube Filter 0.22 μm; Corning, Corning, NY, USA) to increase purity. Collected serum samples were depleted of high-abundant proteins (albumin, antitrypsin, haptoglobin, immunoglobulin A (IgA), immunoglobulin G (IgG), and transferrin) using a multiple-affinity removal system liquid chromatography (LC) column (human 6-HC, 4.6 × 50 mm; Agilent Technologies, Santa Clara, CA, USA). The obtained low-abundant proteins were centrifuged at 12, 000× *g* and 4 °C for 25 min using the Nanosep^®^ centrifugal device (MWCO 3000; Pall Corporation, Port Washington, NY, USA) and dried in ScanSpeed 40 coupled with Teflon (LaboGene, Lillerød, Denmark).

The protein concentration of pooled and individual serum samples for mass spectrometry (MS) analysis was 1 mg and 100 μg, respectively. Subsequently, 5 mM tris (2-carboxyethyl) phosphine (Pierce, Rockford, IL, USA) was added to samples, and samples were reduced at 37 °C for 30 min. Then, 0.5 mM iodoacetamide (Sigma-Aldrich) was added to samples for conducting alkylation under dark conditions at 25 °C for 1 h. MS-grade trypsin gold (Promega, Madison, WI, USA) was added to samples to enable digestion of proteins into peptides, with overnight shaking at 37 °C. C18 cartridges (Waters, Milford, MA, USA) were used to clean the chemical reagents contained in samples. Samples were lyophilized using a ScanSpeed 40 coupled with Teflon.

Finally, 0.1% formic acid (Sigma-Aldrich) in HPLC-grade water was added to dried samples for performing MS analysis. Pooled serum samples were separated into groups of 12 samples according to their isoelectric point using an OFFGEL Fractionator with a 12-well setup (3100 OFFGEL Loss Res Kit, pH 3–10; Agilent Technologies).

### 4.3. MS Analysis Using Mass Spectrometer

Pooled and individual serum samples were analyzed using a Triple TOF 5600 mass spectrometer (AB SCIEX, Concord, ON, Canada) combined with a Nano-LC system Ekspert nLC415 (Eksigent Technologies, Dublin, CA, USA).

To proceed with protein identification, 2 μL from each pooled and individual serum sample containing 1 mg and 100 μg/μL, respectively, was injected into the Eksigent ChromXP nanoLC trap column (3 μm, 350 μm × 0.5 mm; Eksigent Technologies) at a flow rate of 5000 nL/min for 5 min and eluted using an Eksigent ChromXP nanoLC column (3 μm, 75 μm × 150 mm; Eksigent Technologies). Mobile phase A was composed of 0.1% formic acid in HPLC-grade water, whereas mobile phase B was composed of 0.1% formic acid in HPLC-grade acetonitrile (ACN). The flow rate was 300 nL/min, and the mobile phase B gradient was 5–90% for a total of 120 min. The gradient method was performed as follows: (finished time/mobile phase B%) 0 min/5%, 105 min/40%, 105.5 min/90%, 111.5 min/90%, 112 min/5%, and 120 min/5% mobile phase B. After analysis of every three samples, 50 fM β-galactose was used for auto-calibration. The parameters of ion source gas, curtain gas, ion spray voltage floating, and interface heater temperature were 12, 25, 2200 V, and 150 °C, respectively.

### 4.4. IDA Mode Using Pooled Serum Samples

Pooled serum samples were analyzed using the IDA mode to generate a library. The MS range was scanned at 250–2000 mass-to-charge ratio (*m/z*) for precursor ions. The top 10 parent ions with charges ranging from +2 to +5 and intensity >100 cps were selected. Then, the MS/MS range was scanned at 100–2000 *m/z* for product ions. Data from pooled serum samples were obtained using the IDA mode in the ProteinPilot v.5.0 search engine (AB SCIEX) to generate a library.

### 4.5. SWATH Mode Using Individual Serum Samples

Individual serum samples were analyzed for protein identification and relative quantification using the SWATH mode. The MS and MS/MS range were scanned at 250–2500 m/z and 100–2500 *m/z*, for precursor ions and product ions, respectively. The isolation width was 20 Da (containing 1 Da for the window overlap), and a total of 53 overlapping windows were generated.

### 4.6. Protein Identification and Statistical and Functional Analysis for Selection of Protein Candidates

The library produced from pooled serum samples and peak data of individual serum samples were used to identify proteins by matching acquisition mass and retention time of peptides in PeakView v.2.2 (AB SCIEX). The cut-off for the FDR of peptides was <1%, while modified and shared peptides were excluded. Data were normalized using total area sums normalization in MarkerView v.1.3.1 (AB SCIEX), and PCA and *t*-tests were used to identify statistically significant proteins as protein candidates. The cut-offs for *p*-value and fold change (patients with ACS/healthy controls) from the *t*-test were <0.05 and ≥1.2 or ≤1/1.2, respectively. Statistically significant proteins were subjected to functional analysis using the String Database v.11.0 (Search Tool for Retrieval of Interacting Genes/Proteins; https://string-db.org/). Furthermore, we confirmed the accuracy of protein identification through the peak intensity of ≥1000 and FDR of <1% in PeakView and selected the significant proteins. Further, protein candidates for validation through the MRM acquisition mode were selected based on the following criteria: (1) peptides without miscleaved site; (2) modification; (3) M amino acid due to oxidation; and (4) peptides with 7–15 sequence length. Then, two subjects were selected from each group and sex, and proteins satisfying all conditions in all eight selected subjects were identified as the protein candidates.

### 4.7. Validation of Protein Candidates Using the MRM Acquisition Mode for ACS Diagnostic Biomarker Discovery

To proceed with the quantification using the MRM acquisition mode, peptides of the protein candidates with optimal intensity and FDR were selected and synthesized with a purity ≥90% (Peptron, Daejeon, South Korea). The polarity, entrance potential (EP), and collision cell exit potential (CXP) were determined as positive, 10, and 11 V, respectively. Additionally, to set the parameters for quantification, the parameters of collision energy (CE), declustering potential (DP), and retention time were configured for optimal analysis of each synthesized standard peptide via optimization using skyline (http://proteome.gs.washington.edu/software/skyline) and AB SCIEX Triple Quad (QTRAP) 5500 (AB SCIEX). Using only the most suitable parameter for quantification, a standard curve of each standard peptide was generated, and peptides of protein candidates in the sera of subjects were quantified using the AB SCIEX QTRAP 5500 equipped with the ACQUITY UPLC BEH C18 column (130 Å, 1.7 µm, 2.1 mm × 150 mm, Waters) and the ACQUITY UPLC BEH C18 VanGuard pre-column (130 Å, 1.7 µm, 2.1 mm × 5 mm, Waters). The injected 5-μL sample was analyzed using a mobile phase B of 5–90% and 0.25 μL/min flow rate for a total duration of 30 min. Mobile phases A and B consisted of 0.1% formic acid in HPLC grade water and 0.1% formic acid in HPLC grade ACN, respectively. The gradient method was performed as follows: (finished time/mobile phase B%) 0 min/10%, 1 min/15%, 20 min/40%, 21 min/90%, 25 min/90%, 25.5/10%, and 30 min/10% mobile phase B. The parameters used were as follows: ion source gas 1 and 2, curtain gas, ion spray voltage, and temperature were 40, 60, 30, 5, 500 V, and 400 °C, respectively. Results of our analysis were normalized using an internal standard.

The Kolmogorov–Smirnov test was conducted for normalization of quantitative results obtained by utilization of the MRM acquisition mode. The *t*-test or the Mann–Whitney *U* test was performed to confirm the statistical significance, and data have been represented via scatter plot and ROC curve using the GraphPad Prism v.8.4.2 (GraphPad Software, La Jolla, CA, USA).

## Figures and Tables

**Table 1:** List of protein candidates used for validation.

No.	UniProt Accession No.	Protein Name	Expression	*p*-Value	Fold Change
1	P01011	α-1-antichymotrypsin	Up	7.50 × 10^−4^	1.22
2	P10909	Clusterin	Up	2.75 × 10^−5^	1.40
3	P02790	Hemopexin	Up	2.60 × 10^−7^	1.38
4	P02750	Leucine-rich α-2-glycoprotein	Up	1.87 × 10^−6^	1.64
5	P04004	Vitronectin	Up	2.90 × 10^−4^	1.43
6	P02654	Apolipoprotein C-I	Down	1.74 × 10^−2^	0.74
7	P02751	Fibronectin	Down	2.10 × 10^−5^	0.39

**Table 2:** Parameters of the multiple reaction monitoring (MRM) acquisition mode for ACS diagnostic biomarker discovery.

No.	UniProt Accession No.	Protein Name	Peptide Sequence	Target Ion	Q1 (*m/z*)	Q3 (*m/z*)	RT (min)	DP (V)	CE (V)
1	P01011	α-1-antichymotrypsin	ITLLSALVETR	2y4	608.369	504.278	11.56	75.5	30.8
				**2y8**	**608.369**	**888.515**	**11.56**	**75.5**	**30.8**
				3y4	405.915	504.278	11.56	60.7	19.7
2	P10909	Clusterin	LFDSDPITVTVPVEVSR	2y6	937.499	686.383	10.56	99.5	42.6
				2y8	937.499	886.499	10.56	99.5	42.6
				**3y6**	**625.335**	**686.383**	**10.56**	**76.7**	**31.6**
3	P02790	Hemopexin	LWWLDLK	2b2	487.279	300.171	12.81	66.6	26.4
				**2y2**	**487.279**	**260.197**	**12.79**	**66.6**	**26.4**
				2y5	487.279	674.387	12.85	66.6	26.4
4	P02750	Leucine-rich α-2-glycoprotein	DLLLPQPDLR	**2b2**	**590.34**	**229.118**	**8.99**	**74.2**	**30.1**
				2b3	590.34	342.202	9.00	74.2	30.1
				2y6	590.34	725.394	9.00	74.2	30.1
5	P04004	Vitronectin	FEDGVLDPDYPR	2b2	711.83	277.118	5.90	83.0	34.5
				**2y5**	**711.83**	**647.315**	**5.90**	**83.0**	**34.5**
				2y7	711.83	875.426	5.90	83.0	34.5
6	P02654	Apolipoprotein C-I	TPDVSSALDK	**2y6**	**516.764**	**620.325**	**3.20**	**68.8**	**27.5**
				2y7	516.764	719.393	3.19	68.8	27.5
				2y8	516.764	834.420	3.19	68.8	27.5
7	P02751	Fibronectin	SYTITGLQPGTDYK	**2b3**	**772.386**	**352.150**	**5.18**	**87.4**	**36.7**
				2y6	772.386	680.325	5.18	87.4	36.7
				2y10	772.386	1079.537	5.18	87.4	36.7

The target ion indicated in boldface font was used during the final quantification process. Q1, precursor ion; Q3, product ion; RT, retention time; DP, declustering potential; CE, collision energy.

**Table 3:** Demographic information of subjects.

Parameter	Discovery Set		Validation Set	
	Patients With ACS (*n* = 20)	Healthy Controls (*n* = 20)	Patients With ACS (*n* = 50)	Healthy Controls (*n* = 50)
Sex (Female/Male)	9/11	8/12	19/31	24/26
Age ^a^ (Years)	63.1 ± 11.8	54.9 ± 7.8	64.5 ± 11.7	53.8 ± 5.9
BMI ^a^ (kg/m^2^)	24.7 ± 3.5	23.9 ± 3.2	24.6 ± 3.3	23.2 ± 2.6
Blood pressure ^a^ (Systolic/Diastolic mm Hg)	131.0 ± 33.6/80.4 ± 11.1	123.7 ± 16.3/77.1 ± 11.9	128.1 ± 25.1/79.3 ± 13.2	123.5 ± 14.7/76.4 ± 10.5
Smoking (No/Yes)	13/7	10/10	32/18	35/15
Diabetes Mellitus (No/Yes)	11/9	20/0	34/16	49/1
Hyperlipidemia (No/Yes)	9/11	19/1	24/26	50/0
Hypertension (No/Yes)	8/12	20/0	23/27	50/0
Familial CAD (No/Yes)	20/0	17/3	50/0	46/4
Previous CABG (No/Yes)	20/0	N/A	50/0	N/A
Previous PCI (No/Yes)	16/4	N/A	40/10	N/A
LVEF ^a^ (%)	56.8 ± 9.3	N/A	55.1 ± 9.2	N/A
CK-MB ^a^ (ng/mL)	28.1 ± 72.7	N/A	35.9 ± 76.1	N/A
LDL ^a^ (mg/dL)	92.9 ± 30.3	118 ± 32.1	99.3 ± 33.2	118.6 ± 29.7
Triglyceride ^a^ (mg/dL)	129.1 ± 61.9	139.3 ± 74.8	157.9 ± 108.0	109.6 ± 45.0
Troponin T ^a^ (ng/mL)	0.9 ± 2.1	N/A	1.3 ± 2.4	N/A

^a^ mean ± standard deviation; BMI, body mass index; CAD, cardiac artery disease; CABG, coronary artery bypass graft; PCI, percutaneous coronary intervention; LVEF, left ventricular ejection fraction; CK-MB, creatinine kinase-myocardial band; LDL, low-density lipoprotein.

## Data Availability

The data that support the findings of this study are available from the corresponding author upon reasonable request.

## Supplementary Materials

The following supplementary materials are available online, Table S1: list of differentially expressed proteins in patients with ACS and healthy controls, Table S2: list of proteins related to the biological process of functional analysis, Table S3: list of proteins related to the molecular function of functional analysis.

## Sample Availability

Samples are not available from the authors.

## References

[1] Kalra S., Duggal S., Valdez G., Smalligan R.D. (2008). Review of acute coronary syndrome diagnosis and management. Postgrad. Med..

[2] Arroyo L.H., Lee R.T. (1999). Mechanisms of plaque rupture: Mechanical and biologic interactions. Cardiovasc. Res..

[3] Vogel B., Claessen B.E., Arnold S.V., Chan D., Cohen D.J., Giannitsis E., Gibson C.M., Goto S., Katus H.A., Kerneis M. (2019). ST-segment elevation myocardial infarction. Nat. Rev. Dis. Primers.

[4] Waller D.G., Sampson A.P. (2018). Ischaemic heart disease. Medical Pharmacology and Therapeutics.

[5] Eisen A., Giugliano R.P., Braunwald E. (2016). Updates on Acute Coronary Syndrome: A Review. JAMA Cardiol..

[6] McCune C., McKavanagh P., Menown I.B. (2015). A Review of Current Diagnosis, Investigation, and Management of Acute Coronary Syndromes in Elderly Patients. Cardiol. Ther..

[7] Joo J.H., Liao J.M., Bakaeen F.G., Chu D. (2018). Surgical revascularization for acute coronary syndromes: A narrative review. Vessel Plus.

[8] Mueller C. (2014). Biomarkers and acute coronary syndromes: An update. Eur. Heart J..

[9] McCann C.J., Glover B.M., Menown I.B., Moore M.J., McEneny J., Owens C.G., Smith B., Sharpe P.C., Young I.S., Adgey J.A. (2008). Novel biomarkers in early diagnosis of acute myocardial infarction compared with cardiac troponin T. Eur. Heart J..

[10] Crutchfield C.A., Thomas S.N., Sokoll L.J., Chan D.W. (2016). Advances in mass spectrometry-based clinical biomarker discovery. Clin. Proteom..

[11] O’Gara P.T., Kushner F.G., Ascheim D.D., Casey D.E., Chung M.K., de Lemos J.A., Ettinger S.M., Fang J.C., Fesmire F.M., Franklin B.A. (2013). 2013 ACCF/AHA guideline for the management of ST-elevation myocardial infarction: A report of the American College of Cardiology Foundation/American Heart Association Task Force on Practice Guidelines. J. Am. Coll. Cardiol..

[12] Bolatkale M., Acara A.C. (2020). A Novel Index for Prompt Prediction of Severity in Patients with Unstable Angina Pectoris. Emerg. Med. Int..

[13] Betzen C., Alhamdani M.S., Lueong S., Schröder C., Stang A., Hoheisel J.D. (2015). Clinical proteomics: Promises, challenges and limitations of affinity arrays. Proteom. Clin. Appl..

[14] Han X., Aslanian A., Yates J.R. (2008). Mass spectrometry for proteomics. Curr. Opin. Chem. Biol..

[15] Aslam B., Basit M., Nisar M.A., Khurshid M., Rasool M.H. (2017). Proteomics: Technologies and Their Applications. J. Chromatogr. Sci..

[16] Cho W.C.S. (2007). Proteomics Technologies and Challenges. Genom. Proteom. Bioinform..

[17] Park A., Lee J., Mun S., Kim D.J., Cha B.H., Moon K.T., Yoo T.K., Kang H.G. (2017). Identification of Transcription Factor YY1 as a Regulator of a Prostate Cancer-Specific Pathway Using Proteomic Analysis. J. Cancer.

[18] Kim D., Mun S., Lee J., Park A., Seok A., Chun Y.T., Kang H.G. (2018). Proteomics analysis reveals differential pattern of widespread protein expression and novel role of histidine-rich glycoprotein and lipopolysaccharide-binding protein in rheumatoid arthritis. Int. J. Biol. Macromol..

[19] Aebersold R., Mann M. (2003). Mass spectrometry-based proteomics. Nature.

[20] Bhosale S.D., Moulder R., Kouvonen P., Lahesmaa R., Goodlett D.R. (2017). Mass Spectrometry-Based Serum Proteomics for Biomarker Discovery and Validation. Methods Mol. Biol..

[21] Diamandis E.P. (2004). Mass spectrometry as a diagnostic and a cancer biomarker discovery tool: Opportunities and potential limitations. Mol. Cell. Proteom..

[22] Lee J., Mun S., Kim D., Lee Y.R., Sheen D.H., Ihm C., Lee S.H., Kang H.G. (2019). Proteomics Analysis for Verification of Rheumatoid Arthritis Biomarker Candidates Using Multiple Reaction Monitoring. Proteom. Clin. Appl..

[23] Lee J., Mun S., Park A., Kim D., Lee Y.J., Kim H.J., Choi H., Shin M., Lee S.J., Kim J.G. (2020). Proteomics Reveals Plasma Biomarkers for Ischemic Stroke Related to the Coagulation Cascade. J. Mol. Neurosci..

[24] Lam M.P.Y., Ping P., Murphy E. (2016). Proteomics Research in Cardiovascular Medicine and Biomarker Discovery. J. Am. Coll. Cardiol..

[25] Ku E.J., Cho K.-C., Lim C., Kang J.W., Oh J.W., Choi Y.R., Park J.-M., Han N.-Y., Oh J.J., Oh T.J. (2020). Discovery of plasma biomarkers for predicting the severity of coronary artery atherosclerosis by quantitative proteomics. BMJ Open Diabetes Res. Care.

[26] Gao S., Liu J. (2017). Association between circulating oxidized low-density lipoprotein and atherosclerotic cardiovascular disease. Chronic Dis. Transl. Med..

[27] Tousoulis D., Kampoli A.M., Papageorgiou N., Androulakis E., Antoniades C., Toutouzas K., Stefanadis C. (2011). Pathophysiology of atherosclerosis: The role of inflammation. Curr. Pharm. Des..

[28] Lindstedt K.A., Leskinen M.J., Kovanen P.T. (2004). Proteolysis of the Pericellular Matrix. Arterioscler. Thromb. Vasc. Biol..

[29] Jung J.Y., Kwak Y.H., Chang I., Kwon W.Y., Suh G.J., Choi D. (2017). Protective effect of hemopexin on systemic inflammation and acute lung injury in an endotoxemia model. J. Surg. Res..

[30] Tolosano E., Altruda F. (2002). Hemopexin: Structure, function, and regulation. DNA Cell Biol..

[31] Lin T., Sammy F., Yang H., Thundivalappil S., Hellman J., Tracey K.J., Warren H.S. (2012). Identification of hemopexin as an anti-inflammatory factor that inhibits synergy of hemoglobin with HMGB1 in sterile and infectious inflammation. J. Immunol..

[32] Alfakry H., Malle E., Koyani C.N., Pussinen P.J., Sorsa T. (2016). Neutrophil proteolytic activation cascades: A possible mechanistic link between chronic periodontitis and coronary heart disease. Innate Immun..

[33] Mehta N.U., Grijalva V., Hama S., Wagner A., Navab M., Fogelman A.M., Reddy S.T. (2016). Apolipoprotein E-/- Mice Lacking Hemopexin Develop Increased Atherosclerosis via Mechanisms That Include Oxidative Stress and Altered Macrophage Function. Arterioscler. Thromb. Vasc. Biol..

[34] Stefanic P., Sihotsky V., Hertelyova Z., Kopolovets I., Mathews A.J., Toth S., Kubikova M., Svajdler P., Mucha R., Vasko L. (2019). Interleukin-4, hemopexin, and lipoprotein-associated phospholipase A2 are significantly increased in patients with unstable carotid plaque. Open Chem..

[35] Yalamanoglu A., Deuel J.W., Hunt R.C., Baek J.H., Hassell K., Redinius K., Irwin D.C., Schaer D.J., Buehler P.W. (2018). Depletion of haptoglobin and hemopexin promote hemoglobin-mediated lipoprotein oxidation in sickle cell disease. Am. J. Physiol. Lung Cell Mol. Physiol..

[36] Cubedo J., Suades R., Padro T., Martin-Yuste V., Sabate-Tenas M., Cinca J., Sans-Rosello J., Sionis A., Badimon L. (2017). Erythrocyte-heme proteins and STEMI: Implications in prognosis. Thromb. Haemost..

[37] Kumagai S., Nakayama H., Fujimoto M., Honda H., Serada S., Ishibashi-Ueda H., Kasai A., Obana M., Sakata Y., Sawa Y. (2016). Myeloid cell-derived LRG attenuates adverse cardiac remodelling after myocardial infarction. Cardiovasc. Res..

[38] Yang F.J., Hsieh C.Y., Shu K.H., Chen I.Y., Pan S.Y., Chuang Y.F., Chiu Y.L., Yang W.S. (2020). Plasma Leucine-Rich α-2-Glycoprotein 1 Predicts Cardiovascular Disease Risk in End-Stage Renal Disease. Sci. Rep..

[39] Yang Y., Luo R., Cheng Y., Liu T., Dai W., Li Y., Ge S., Xu G. (2020). Leucine-rich α2-glycoprotein-1 upregulation in plasma and kidney of patients with lupus nephritis. BMC Nephrol..

[40] Saito K., Tanaka T., Kanda H., Ebisuno Y., Izawa D., Kawamoto S., Okubo K., Miyasaka M. (2002). Gene Expression Profiling of Mucosal Addressin Cell Adhesion Molecule-1^+^ High Endothelial Venule Cells (HEV) and Identification of a Leucine-Rich HEV Glycoprotein as a HEV Marker. J. Immunol..

[41] Naka T., Fujimoto M. (2018). LRG is a novel inflammatory marker clinically useful for the evaluation of disease activity in rheumatoid arthritis and inflammatory bowel disease. Immunol. Med..

[42] Ngan D.A., Vickerman S.V., Granville D.J., Man S.F.P., Sin D.D. (2009). The possible role of granzyme B in the pathogenesis of chronic obstructive pulmonary disease. Ther. Adv. Respir. Dis..

[43] Xuan C., Li H., Li L.L., Tian Q.W., Wang Q., Zhang B.B., Guo J.J., He G.W., Lun L.M. (2019). Screening and Identification of Pregnancy Zone Protein and Leucine-Rich Alpha-2-Glycoprotein as Potential Serum Biomarkers for Early-Onset Myocardial Infarction using Protein Profile Analysis. Proteom. Clin. Appl..

[44] Yaghoubi A., Ghojazadeh M., Abolhasani S., Alikhah H., Khaki-Khatibi F. (2015). Correlation of Serum Levels of Vitronectin, Malondialdehyde and Hs- CRP With Disease Severity in Coronary Artery Disease. J. Cardiovasc. Thorac. Res..

[45] Aslan S., Ikitimur B., Cakmak H.A., Karadag B., Tufekcioglu E.Y., Ekmekci H., Yuksel H. (2015). Prognostic utility of serum vitronectin levels in acute myocardial infarction. Herz.

[46] Zhong J., Yang H.-C., Kon V., Fogo A.B., Lawrence D.A., Ma J. (2014). Vitronectin-binding PAI-1 protects against the development of cardiac fibrosis through interaction with fibroblasts. Lab. Investig..

[47] Konstantinides S., Schäfer K., Thinnes T., Loskutoff D.J. (2001). Plasminogen Activator Inhibitor-1 and Its Cofactor Vitronectin Stabilize Arterial Thrombi After Vascular Injury in Mice. Circulation.

[48] Lee C.-C., Huang T.-S. (2005). Plasminogen activator inhibitor-1: The expression, biological functions, and effects on tumorigenesis and tumor cell adhesion and migration. J. Cancer Mol..

[49] Ekmekci H., Sonmez H., Ekmekci O.B., Ozturk Z., Domanic N., Kokoglu E. (2002). Plasma vitronectin levels in patients with coronary atherosclerosis are increased and correlate with extent of disease. J. Thromb. Thrombolysis.

[50] Raghunath P.N., Tomaszewski J.E., Brady S.T., Caron R.J., Okada S.S., Barnathan E.S. (1995). Plasminogen Activator System in Human Coronary Atherosclerosis. Arterioscler. Thromb. Vasc. Biol..

[51] Al-Yafeai Z., Yurdagul A., Peretik J.M., Alfaidi M., Murphy P.A., Orr A.W. (2018). Endothelial FN (Fibronectin) Deposition by alpha5beta1 Integrins Drives Atherogenic Inflammation. Arterioscler. Thromb. Vasc. Biol..

[52] Dai R., Iwama A., Wang S., Kapila Y.L. (2005). Disease-associated fibronectin matrix fragments trigger anoikis of human primary ligament cells: p53 and c-myc are suppressed. Apoptosis.

[53] Dollery C.M., Libby P. (2006). Atherosclerosis and proteinase activation. Cardiovasc. Res..

[54] Yamac A.H., Sevgili E., Kucukbuzcu S., Nasifov M., Ismailoglu Z., Kilic E., Ercan C., Jafarov P., Uyarel H., Bacaksiz A. (2015). Role of cathepsin D activation in major adverse cardiovascular events and new-onset heart failure after STEMI. Herz.

[55] Velez P., Parguina A.F., Ocaranza-Sanchez R., Grigorian-Shamagian L., Rosa I., Alonso-Orgaz S., de la Cuesta F., Guitian E., Moreu J., Barderas M.G. (2014). Identification of a circulating microvesicle protein network involved in ST-elevation myocardial infarction. Thromb. Haemost..

[56] Rathnayake N., Gustafsson A., Norhammar A., Kjellström B., Klinge B., Rydén L., Tervahartiala T., Sorsa T., Group P.S. (2015). Salivary Matrix Metalloproteinase-8 and -9 and Myeloperoxidase in Relation to Coronary Heart and Periodontal Diseases: A Subgroup Report from the PAROKRANK Study (Periodontitis and Its Relation to Coronary Artery Disease). PLoS ONE.

[57] Salminen A., Åström P., Metso J., Soliymani R., Salo T., Jauhiainen M., Pussinen P.J., Sorsa T. (2015). Matrix metalloproteinase 8 degrades apolipoprotein A-I and reduces its cholesterol efflux capacity. Faseb J..

