# Supplementary material for: Biomarker Discovery of Acute Coronary Syndrome Using Proteomic Approach
Source: Molecules. 2021 Feb 20;26(4):1136. doi: 10.3390/molecules26041136 (PMC7924321; doi:10.3390/molecules26041136)
Supplement: Supplementary file 1 [file molecules-26-01136-s001.pdf]

Supplementary Table S1. List of differentially expressed proteins in patients with acute coronary syndrome (ACS) and healthy controls.

No.	UniProt accession no.	Protein name	Abbreviation of protein name	Expression	*p*-value	Fold change
1	P02763	α-1-acid glycoprotein 1	AGP1	Up	1.00.E^{-04}	1.65
2	P19652	α-1-acid glycoprotein 2	AGP2	Up	5.52.E^{-03}	1.33
3	P01011	α-1-antichymotrypsin	ACT	Up	7.50.E^{-04}	1.22
4	O75882	Attractin	ATRN	Up	5.30.E^{-04}	1.31
5	P00915	Carbonic anhydrase 1	CA1	Up	4.70.E^{-02}	1.61
6	P10909	Clusterin	CLU	Up	2.75.E^{-05}	1.40
7	P02746	Complement C1q subcomponent subunit B	C1QB	Up	9.70.E^{-04}	1.26
8	P00736	Complement C1r subcomponent	C1R	Up	2.03.E^{-03}	1.22
9	P0C0L4	Complement C4-A	C4A	Up	3.20.E^{-03}	1.37
10	P0C0L5	Complement C4-B	C4B	Up	1.09.E^{-03}	1.46
11	P01031	Complement C5	C5	Up	2.67.E^{-07}	1.37
12	P13671	Complement component C6	C6	Up	1.99.E^{-03}	1.24
13	P07360	Complement component C8 γ chain	C8G	Up	2.93.E^{-03}	1.63
14	P02748	Complement component C9	C9	Up	3.62.E^{-06}	1.58
15	P08603	Complement factor H	CFH	Up	7.44.E^{-08}	1.56
16	P05156	Complement factor I	CFI	Up	9.90.E^{-06}	1.29
17	P02790	Hemopexin	HPX	Up	2.60.E^{-07}	1.38
18	P04196	Histidine-rich glycoprotein	HRG	Up	8.30.E^{-04}	2.10
19	Q06033	Inter-α-trypsin inhibitor heavy chain H3	ITIH3	Up	4.65.E^{-06}	1.87
20	Q14624	Inter-α-trypsin inhibitor heavy chain H4	ITIH4	Up	2.86.E^{-09}	1.32
21	P02750	Leucine-rich α-2-glycoprotein	LRG	Up	1.87.E^{-06}	1.64
22	P36955	Pigment epithelium-derived factor	PEDF	Up	1.24.E^{-03}	1.23
23	P02760	Protein AMBP	AMBP	Up	2.00.E^{-04}	1.75
24	Q9UK55	Protein Z-dependent protease inhibitor	ZPI	Up	5.29.E^{-03}	1.29
25	P02743	Serum amyloid P-component	SAP	Up	2.65.E^{-03}	1.47
26	P05543	Thyroxine-binding globulin	TBG	Up	8.40.E^{-04}	1.34
27	P02774	Vitamin D-binding protein	DBP	Up	1.42.E^{-02}	1.23
28	P04004	Vitronectin	VN	Up	2.90.E^{-04}	1.43
29	P25311	Zinc-α-2-glycoprotein	ZAG	Up	2.10.E^{-05}	1.38
30	P02765	α-2-HS-glycoprotein	AHSG	Down	4.04.E^{-05}	0.63
31	P02647	Apolipoprotein A-I	ApoA-I	Down	4.46.E^{-14}	0.76
32	P02652	Apolipoprotein A-II	ApoA-II	Down	3.75.E^{-02}	0.82
33	P02654	Apolipoprotein C-I	ApoC-I	Down	1.74.E^{-02}	0.74
34	O14791	Apolipoprotein L1	ApoL1	Down	5.05.E^{-03}	0.69
35	O95445	Apolipoprotein M	ApoM	Down	3.27.E^{-02}	0.82
36	P08185	Corticosteroid-binding globulin	CBG	Down	1.33.E^{-02}	0.72
37	Q16610	Extracellular matrix protein 1	ECM1	Down	1.70.E^{-02}	0.76
38	P02751	Fibronectin	FN	Down	2.10.E^{-05}	0.39
39	P06396	Gelsolin	GSN	Down	5.03.E^{-05}	0.75
40	P35542	Serum amyloid A-4 protein	SAA4	Down	3.58.E^{-03}	0.77
41	P05452	Tetranectin	TN	Down	1.84.E^{-03}	0.79
42	P02766	Transthyretin	TTR	Down	1.34.E^{-03}	0.79

Supplementary Table S2. List of proteins related to the biological process of functional analysis.

No.	Biological process	Abbreviation of protein name
1	Regulation of proteolysis	ACT, AHSG, AMBP, C1QB, C1R, C4A, C4B, C5, C6, C8G, C9, CBG, CFH, CFI, CLU, ECM1, FN, GSN, HRG, ITIH3, ITIH4, PEDF, TBG, TN, VN, ZPI
2	Regulation of humoral immune response	C1QB, C1R, C4A, C4B, C5, C6, C8G, C9, CFH, CFI, CLU, HPX, VN
3	Regulation of complement activation	C1QB, C1R, C4A, C4B, C5, C6, C8G, C9, CFH, CFI, CLU, VN
4	Regulation of protein processing	C1QB, C1R, C4A, C4B, C5, C6, C8G, C9, CFH, CFI, CLU, GSN, TN, VN
5	Regulation of protein activation cascade	C1QB, C1R, C4A, C4B, C5, C6, C8G, C9, CFH, CFI, CLU, VN
6	Negative regulation of hydrolase activity	ACT, AHSG, AMBP, ApoA-I, ApoA-II, ApoC-I, C4A, C4B, C5, CBG, ECM1, HRG, ITIH3, ITIH4, PEDF, SAP, TBG, VN, ZPI
7	Regulation of acute inflammatory response	C1QB, C1R, C4A, C4B, C5, C6, C8G, C9, CFH, CFI, CLU, SAP, VN
8	Defense response	ACT, AHSG, AGP1, AGP2, ApoA-II, ApoL1, ATRN, C1QB, C1R, C4A, C4B, C5, C6, C8G, C9, CFH, CFI, CLU, ECM1, FN, GSN, HRG, ITIH4, SAA4, SAP
9	Complement activation	C1QB, C1R, C4A, C4B, C5, C6, C8G, C9, CFH, CFI, CLU
10	Complement activation, classical pathway	C1QB, C1R, C4A, C4B, C5, C6, C8G, C9, CFI, CLU

Supplementary Table S3. List of proteins related to the molecular function of functional analysis.

No.	Biological process	Abbreviation of protein name
1	Peptidase regulator activity	ACT, AHSG, AMBP, C4A, C4B, C5, CBG, FN, HRG, ITIH3, ITIH4, PEDF, TBG, ZPI
2	Endopeptidase inhibitor activity	ACT, AHSG, AMBP, C4A, C4B, C5, CBG, HRG, ITIH3, ITIH4, PEDF, TBG, ZPI
3	Enzyme inhibitor activity	ACT, AHSG, AMBP, ApoA-I, ApoA-II, ApoC-I, C4A, C4B, C5, CBG, HRG, ITIH3, ITIH4, PEDF, TBG, ZPI
4	Serine-type endopeptidase inhibitor activity	ACT, AMBP, CBG, HRG, ITIH3, ITIH4, PEDF, TBG, ZPI
5	Enzyme regulator activity	ACT, AHSG, AMBP, ApoA-I, ApoA-II, ApoC-I, C4A, C4B, C5, FN, HRG, ITIH3, ITIH4, ZPI, CBG, TBG, PEDF
6	Molecular function regulator	AHSG, AMBP, ApoA-I, ApoA-II, ApoC-I, C4A, C4B, C5, CBG, FN, ACT, HRG, ITIH3, ITIH4, SAA4, PEDF, TBG, TTR, ZPI
7	Complement binding	C4A, C4B, C8G, SAP
8	Phosphatidylcholine-sterol O-acyltransferase activator activity	ApoA-I, ApoA-II, ApoC-I
9	Heparin binding	CFH, FN, TN, HRG, VN, ZPI
10	Lipase inhibitor activity	ApoA-I, ApoA-II, ApoC-I
